# Feasibility of a home-based foot–ankle exercise programme for musculoskeletal dysfunctions in people with diabetes: randomised controlled FOotCAre (FOCA) Trial II

**DOI:** 10.1038/s41598-021-91901-0

**Published:** 2021-06-11

**Authors:** Érica Q. Silva, Danilo P. Santos, Raquel I. Beteli, Renan L. Monteiro, Jane S. S. P. Ferreira, Ronaldo H. Cruvinel-Junior, Asha Donini, Jady L. Verissímo, Eneida Y. Suda, Isabel C. N. Sacco

**Affiliations:** 1 Department of Physical Therapy, Speech, and Occupational Therapy, Faculdade de Medicina, Universidade de São Paulo, Rua Cipotânea, 51, Cidade Universitária, São Paulo, SP 05360-160 Brazil; 2 Department of Physical Therapy, Universidade Federal do Amapá, Amapá, Brazil; 3 Department of Physical Therapy, Universidade Ibirapuera, São Paulo, SP Brazil

**Keywords:** Endocrine system and metabolic diseases, Neurological manifestations, Disease prevention, Patient education, Quality of life

## Abstract

This study sought to assess the feasibility of design, adherence, satisfaction, safety and changes in outcomes followed by a home-based foot–ankle exercise guided by a booklet in individuals with diabetic peripheral neuropathy (DPN). 20 participants were allocated usual care [control group (CG)] or usual care plus home-based foot–ankle exercises [intervention group (IG)] for 8 weeks. For feasibility, we assessed contact, preliminary screening and recruitment rates, adherence, and using a 5-point Likert scale to satisfaction and safety of the booklet. In the IG, we assessed preliminary changes in DPN symptoms, DPN severity (classified by a fuzzy model) and foot–ankle range of motion between baseline and Week 8. In the first 20 weeks, 1310 individuals were screened for eligibility by phone contact. Contact rate was 89% (contacted participants/20w), preliminary screening success 28% (participants underwent screening/20w), and recruitment rate 1.0 participants/week (eligible participants/20w). The recruitment rate was less than the ideal rate of 5 participants/week. The adherence to the exercises programme was 77%, and the dropout was 11% and 9% for the IG and CG, respectively. In the IG, participants’ median level of satisfaction was 4 (IQR: 4–5) and perceived safety was 3 (IQR: 3–5). IG significantly decreased the DPN severity (p = 0.020), increased hallux relative to forefoot (first metatarsal) range of motion (ROM) (p < 0.001) and decreased maximum forefoot relative to hindfoot (midfoot motion) dorsiflexion during gait (p = 0.029). The home-based programme was feasible, satisfactory, safe and showed preliminary positive changes in DPN severity and foot motion during gait.

**Trial Registration** ClinicalTrials.gov, NCT04008745. Registered 02/07/2019. https://clinicaltrials.gov/ct2/show/NCT04008745.

## Introduction

Diabetic peripheral neuropathy (DPN) is the most common chronic complication of diabetes affecting up to 50% of the diabetic population, which has as a major outcome the formation of ulcers and amputation^1^. Complications including progressive loss of vibratory, thermal, tactile and proprioception sensitivities^2^; loss of motor units and motor axons^3^; skin breakdown^4^; and lower limb musculoskeletal complications disrupt the daily living activities and quality of life of people with diabetes^5^. Musculoskeletal complications include muscles and locomotor alterations, such as progressive atrophy of the foot–ankle extrinsic and intrinsic muscles leading to common foot deformities^6^; an increased proportion of fat tissue within foot muscles^7^ and altered mechanical properties of the calcaneal tendon^8^. Altered locomotion mechanics include impairments in the lower limbs’ muscle activation magnitude and timing^9,10^; decreased foot–ankle range of motion (ROM)^6,11^; high plantar pressure and changes in foot rollover temporal parameters^12,13^.

Therapeutic supervised exercises targeting the foot–ankle may be beneficial for people with DPN and were recommended for the first time in the most recent guidelines of the International Working Group on the Diabetic Foot^14^. It has been shown that supervised therapeutic foot–ankle exercises improve DPN signs and symptoms^15–18^; foot rollover and pressure-related variables while walking^17,19–22^; gait temporal parameters^21,23,24^; foot–ankle muscle strength^16,23^ and foot–ankle joint mobility^17,25^. However, not all positive effects are retained at follow-up^26,27^ suggesting the need for a continuous educational process for improving self-management and self-care.

Home-based exercise/rehabilitation is a self-management approach for people with chronic diseases. For most of this population, self-management is a lifetime taskl^28^. Recent clinical trials and reviews have shown that home-based exercises are safe, have a good compliance and presents efficacy for chronic diseases and conditions, such as cancer^29^, Parkinson’s disease^30^, cardiac and pulmonary diseases^31^, dementia^32^, stroke^33^ and complications of old age^34^. Although we did not find any evidence for the efficacy of a home-based exercise programme focusing on foot–ankle deficits in people with diabetes and DPN, Dadgostar et al.^35^ found positive results using an educational booklet that included resistance and aerobic exercises in people with diabetes. However, the general adherence of people with diabetes to exercise is poor^36^, which may compromise the efficacy of a home-based autonomous programme. Therefore, assessing the adherence of people with diabetes and DPN to a home-based, booklet-guided foot exercises programme prior to the development of a larger trial is pivotal because the efficacy of a therapeutic intervention is dependent on participants’ compliance.

Physiotherapy interventions using foot–ankle exercise have not yet been implemented worldwide to prevent the progression of musculoskeletal deficits in people with diabetes and DPN. For this reason, it is still unclear whether recruitment for a trial that tests the efficacy of these physiotherapy programmes would be feasible in this population. Thus, the feasibility of a preventive home-based therapeutic programme should first be studied to determine the best training progression in terms of exercises, frequency and intensity and the outcomes used to assess the success of the intervention.

At present, we are conducting a single-blinded, randomised controlled superiority trial with two parallel arms called the FOotCAre (FOCA) trial II to study the efficacy of an 8-week home-based therapeutic foot–ankle exercise programme using an educational booklet in people with DPN. The present study is a first step to investigating the feasibility of the trial in terms of contact, preliminary screening and recruitment rates, adherence to the home-based programme, participant safety and satisfaction and estimated preliminary changes in clinical aspects of DPN and biomechanical outcomes during gait. Our hypotheses are as follows: (H1) the programme will be feasible, (H2) the home-based intervention will show preliminary changes in the DPN symptoms, DPN severity and foot–ankle ROM during gait after 8 weeks.

## Results

### Feasibility outcomes

**Figure 1:**
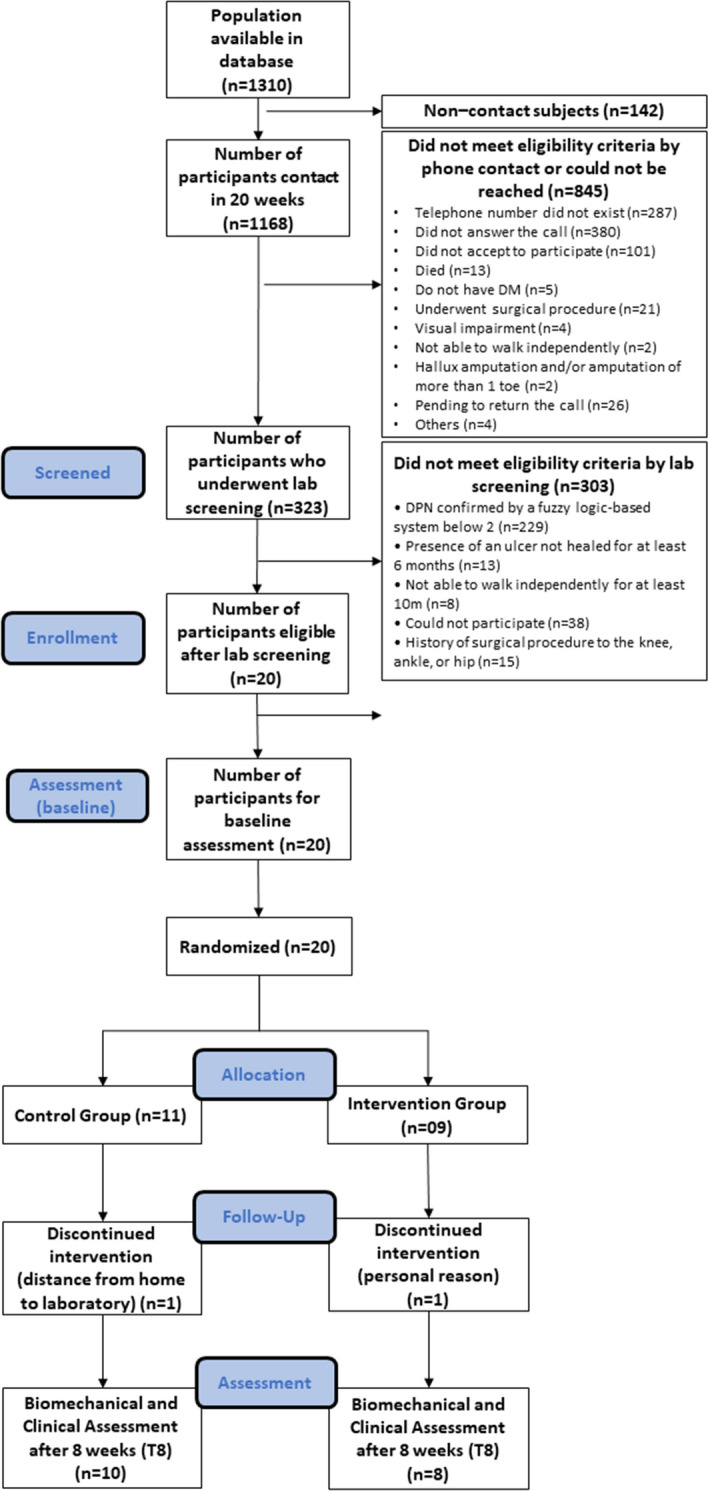
Flow chart of the feasibility study explaining reasons for inclusion and exclusion. *T8* evaluation at 8 weeks, *DM* diabetes mellitus, *DPN* diabetic peripheral neuropathy.

Recruitment was collected from April 23th to September 5th of 2019, totalling 20-week period from a database of 1310 individuals with diabetes mellitus, who were screened for eligibility by phone contact. We were not able to contact 142 candidates (11%) before the recruitment period ended, resulting in a contact rate of 89%. From the 1168 contacted individuals, 323 (28%) met the eligibility criteria as determined over the phone and were scheduled for further screening at the laboratory. Thus, the preliminary screening success was 28%. Further screening of the remaining 323 individuals showed that only 20 (6.2%) had moderate DPN (fuzzy score ≥ 2) and were thus eligible for the study, resulting in a recruitment rate of 1.0 participants/week. Twenty eligible individuals (17 female) were included in the baseline assessment. The challenges of the recruitment process and more details about participant recruitment are described in Fig. 1.

Adherence to the home-based programme was 77.7%, i.e., seven participants out of 9 completed more than 75% of the 24 sessions (at least 18 sessions), and five complete all 24 sessions. The mean (SD) number of sessions performed by all participants that completed the programme was 21.6 (3.5). Of the 20 participants enrolled, two (one each from the CG and IG) dropped out before the T8 assessment, resulting in dropout rates of 9.1% (CG) and 11.1% (IG), respectively. The reasons for dropout were personal reasons and distance from home to the laboratory.

The satisfaction with the programme was excellent to great, with a median score of 4 (IQR: 4–5). Overall, 44.1% considered the programme to be excellent (score of 5 on the Likert scale), 51.9% great (score of 4) and 11.1% good (score of 3). None of the participants rated the programme as poor (score of 2) or terrible (score of 1). For question 7, ‘Would you recommend using the booklet to others with diabetes?’, all participants answered ‘for sure’ (score of 5). Participants considered the booklet to be reasonably safe, with a median score of 3 (IQR: 3–5). No adverse events were reported.

**Figure 2:**
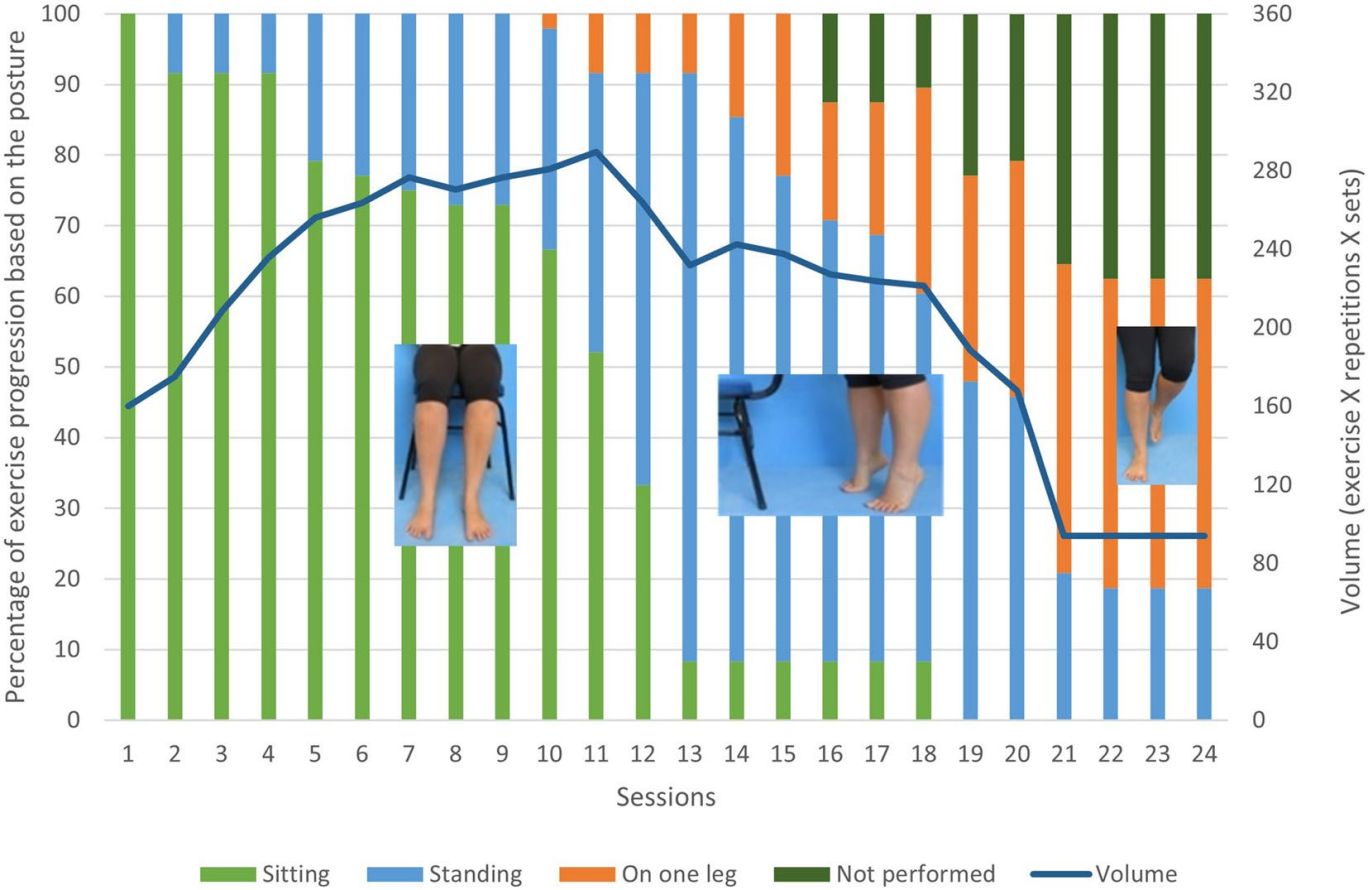
Exercise dose. The general exercise progression from all IG participants from each session (columns) and the volume (repetitions) (blue line) of total workout exercises.

Most of the participants performed the first 10 sessions while sitting, and the volume progressively increased, achieving its peak during session 11 (mean volume = 290) (Fig. 2). The progression of the exercises based on the posture adopted decreased between the sessions 11 and 13. For these sessions, 90% of the participants performed all exercises standing on both legs and 10% standing on one leg, but the overall volume was reduced by 20%, probably because of the increased intensity caused by the posture adopted. In the last four sessions, almost all participants performed all exercises on one leg, and the overall mean volume was 94. The mean number of repetitions performed for all exercises was 25.0 ± 4.5.

### Changes in clinical and biomechanical outcomes

**Figure 3:**
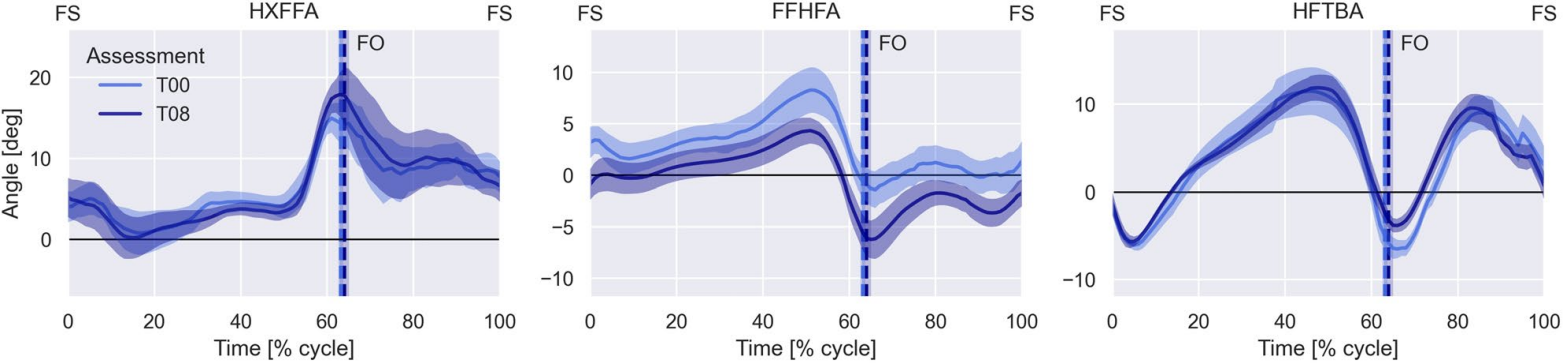
Ensemble averaged kinematics of the hallux (HXFFA), forefoot (FFHFA) and hindfoot (HFTBA) joints in degrees (deg) relative to the proximal segment from de IG participants. Royal blue color indicates baseline assessment (T00), dark blue color indicates Week 8 assessment (T08). Error bars represent standard error. FS indicates foot strike, vertical lines (FO) indicate foot off event in the gait cycle.

At the baseline, the groups were similar for all characteristics and outcomes assessed (Table 1). After 8 weeks, we observed a decrease in DPN severity (fuzzy score), increase in the hallux relative to forefoot (first metatarsal joint) ROM and decrease in the maximum forefoot relative to hindfoot (midfoot motion) dorsiflexion during gait in the IG. There was also an increase trend in the maximum hallux dorsiflexion relative to forefoot (first metatarsal joint), but the difference was not statistically significant (Table 2; Fig. 3).

**Table 1:** Baseline participant’s characteristics from the Control and Intervention groups.

	Control Group (n = 11)	Intervention Group (n = 9)
Age (years)	55.3 (8.9)	58.1 (3.6)
Body mass (kg)	81.0 (15.7)	77.6 (14.7)
Height (cm)	163.5 (0.1)	159.3 (0.1)
Body mass index (kg/m^2^)	29.4 (3.1)	27.6 (12.4)
Sex (male/ female)	(M = 2/F = 9)	(M = 1/F = 8)
Type 2 diabetes (number of participant, %)	8 (72.7%)	7 (77.8%)
Duration of diabetes (years)	20.3 (9.9)	19.1 (11.9)
DPN symptoms (MNSI score)	6.8 (2.8)	4.6 (1.4)
DPN severity (fuzzy score)	5.2 (2.9)	3.6 (2.5)
Tactile sensitivity (number of areas, median [IQR])	2 [0 5]	0 [0 0]
Vibration sensitivity present (number of participants, %)	10 (45%)	13 (72%)
Vibration sensitivity absent (number of participants, %)	7 (32%)	3 (17%)
Vibration sensitivity reduced (number of participants, %)	5 (23%)	2 (11%)
FHSQ—foot pain (score)	31.25 (32.95)	53.75 (26.38)
FHSQ—foot function (score)	56.25 (30.53)	68.75 (20.09)
FHSQ—shoes (score)	16.67 (38.55)	16.67 (28.23)
FHSQ—foot health (score)	25 (26.94)	25 (13.76)

Data are presented as mean (SD) or as n or %. *FHSQ* Foot Health Status Questionnaire, *DPN* diabetic peripheral neuropathy, *MNSI* Michigan Neuropathy Screening Instrument.

**Table 2:** Clinical outcomes and foot–ankle kinematics during gait of the intervention group and within-group difference (baseline to Week 8 (T8)).

Outcomes	Intervention group		Changes in outcomes
	Baseline (n = 9)	T8 (n = 8)	Difference (95% CI)
DPN symptoms (mean MNSI score)	4.6 (1.4)	4.9 (2.4)	0.3 (− 1.3 to 0.5)
DPN severity (fuzzy score)	3.6 (2.5)	2.8 (2.0)	− 0.8 (0.2 to 1.5)
Hallux relative to forefoot dorsiflexion peak (degree)	17.3 (3.4)	20.5 (5.1)	− 3.2 (− 6.4 to 0.07)
Hallux relative to forefoot dorsiflexion ROM (degree)	19.4 (1.5)	23.2 (3.2)	− 3.2 (− 5.1 to -2.1)
Forefoot relative to hindfoot dorsiflexion peak (degree)	11.3 (4.9)	6.3 (2.2)	5.0 (6.7 to 9.3)
Forefoot relative to hindfoot dorsiflexion ROM (degree)	11.9 (3.6)	11.4 (2.8)	− 0.5 (− 3.1 to 4.1)
Hindfoot relative to tibia dorsiflexion peak (degree)	9.4 (5.9)	10.7 (2.3)	1.4 (− 6.7 to 3.9)
Hindfoot relative to tibia dorsiflexion ROM (degree)	16.7 (5.2)	17.5 (1.4)	0.8 (− 4.7 to 3.1)

Data are presented as mean (SD) and differences with 95% confidence intervals. *ROM* range of motion, *DPN* diabetic peripheral neuropathy, *MNSI* Michigan Neuropathy Screening Instrument.

## Discussion

This feasibility study provides preliminary data from a limited sample of DPN participants to determine contact, preliminary screening and recruitment rates, adherence, satisfaction and safety of a home-based intervention.

The recruitment rate was far below laboratory capacity and what was initially anticipated. However, the recruitment of 20 participants in this study was reasonable for starting the RCT. The primary challenge to recruitment was the inclusion criteria. In addition to the age range and health conditions, participants had to have a score ≥ 2 for DPN severity. Mattishent et al.^37^ found a similar low recruitment rate in their feasibility study of individuals with diabetes. Based on this result, new strategies for maximising recruitment are needed. We are developing other strategies, such as advertising on social media and in public places (e.g., metro and bus stations) that this population frequents and broadening our recruitment centres to include primary care centres and secondary and tertiary hospitals. We are continuing to contact potential participants by telephone because this significantly increases recruitment success^38^.

Adherence to the home-based exercise programme exceeded expectations. This could be related to the high satisfaction with the programme, safety of the programme and absence of adverse events. Home-based exercise programmes usually result in a high compliance for several chronic diseases and in older adult populations^29–34^. Supportive elements incorporated into home-based designs, like printed materials, exercise equipment, telephone support, home visits and technology, can be utilised alone or in combination to encourage adherence^39^. Some of the reasons given by our participants for the high compliance and satisfaction and low dropout were the possibility of performing the exercises at home at any time (convenience), thus avoiding using crowded public transportation or heavy traffic. The programme was built to offer autonomy to the participant, with no need for supervision from a health professional when performing the exercises. Considering the high satisfaction and adherence, we feel confident that the purpose of the booklet was successfully achieved.

A low dropout rate among the IG was achieved (11%), which differs from other behavioural trials involving participants with diabetes. In a systematic review, Trivedi et al.^40^ had a mean retention rate of 77.5% (range 36–100%), defined as the ratio between the number of participants retained at follow-up and the number of participants enrolled. Our retention rate of 89% is highly superior.

The exercise volume in each session and progression were acceptable. After 8 weeks of intervention, approximately 95% of the participants who continued to perform the exercises were able to progress to the maximum level of difficulty. However, there was a decrease in the volume of sessions, which could be explained by the progressive increase in the intensity (body posture) of the exercises. In rehabilitation interventions, decreasing the volume and increasing the intensity are fundamental for gaining muscle strength, which is one of the objectives of the full RCT. Thus, this feasibility study showed that was not necessary to modify the initial exercise protocol included in the booklet.

The preliminary changes in outcomes should be interpreted with caution. The DPN severity decreased in the IG, representing an improvement in clinical aspects of DPN. The educational aspects of the programme may have contributed to changing the participants’ health care habits, resulting in better control of diabetes and thus DPN symptoms^41^. Our results corroborate with Sartor et al.^16^, who showed a reduction in DPN symptoms after 12 weeks of a foot–ankle exercise programme. Another aspect that may have contributed in the improvement in DPN severity was subtle changes in the tactile and vibratory sensitivity, which are accounted for the fuzzy score calculation. Kanchanasamut and Pensri^17^ showed changes in vibration perception after 8 weeks of weight-bearing exercise, and Chang et al.^18^ showed improvements in DPN symptoms after 1 year of a Buerger exercise programme.

The intervention improved first metatarsophalangeal joint ROM in the sagittal plane in late stance. A difference of 3.2 degrees was observed, suggesting an improved mobility at the propulsion phase. A greater metatarsophalangeal joint extension may be associated with metatarsophalangeal joint deformity^42^ and may increase the risk of metatarsal heads exposure on the plantar surface repeatedly during gait, increasing plantar pressures at the forefoot^43^. However, our result can be considered positive because according to Deschamps et al.^44^, individuals with DPN present significantly smaller sagittal ROM of the first metatarsophalangeal joint during the forefoot push-off phase. We believe that the increased ROM in this joint is functional, as it was not accompanied by any increase in plantar pressures at the forefoot (will be measured in the full RCT), but it definitely increased the motion of the impaired joint. In addition to the hallux motion, the forefoot dorsiflexion peak was reduced after the intervention, suggesting an increased stiffness in the midfoot excursion during propulsion. The exercises focused on strengthening of the intrinsic foot muscles, such as quadratus plantaris, related to the talonavicular and calcaneocuboid joints. This can lead to increased stiffness of the midfoot during push-off, favouring the midtarsal joint locking mechanism, resulting in a changeover from a flexible foot during weight acceptance to a rigid foot structure during propulsion^45^.

This study’s strengths include its rigorous RCT methodology; customised physiotherapeutic approach guided by a booklet, focusing autonomy of the participant; high satisfaction and adherence rates; and low dropout rate. This is the first study to evaluate the clinical, functional and biomechanical outcomes specifically related to DPN losses using a specialised foot-training protocol guided by a booklet.

This study was limited because participants’ glycaemic and glycated haemoglobin levels were not controlled, so their oscillations may have influenced DPN symptoms. The recruitment strategy needs to be adjusted for the larger trial^46^, and amendment to the original protocol needs to performed. Because this was the only adjustment in the larger trial, the data from participants in this feasibility study will not be excluded from the final analysis.

## Conclusion

This feasibility study showed that the proposed foot–ankle home-based exercise programme is feasible based on the high adherence, satisfaction and perceived safety of the programme, although recruitment strategies need to be improved. The programme showed positive preliminary changes in DPN severity and hallux and forefoot motion during gait, which encourages the development of the larger RCT^46^.

## Methods

### Study design

This feasibility study was part of a series of two clinical trials—the FOot CAre (FOCA) trial I (SOPeD intervention) and FOCA trial II (booklet intervention) and its reporting was based on the Consolidated Standards of Reporting Trials (CONSORT) 2010: extension to randomised pilot and feasibility trials. The trial was approved by the research ethics committee of the School of Medicine of the University of Sao Paulo (CAAE: 90331718.4.0000.0065) and was registered at ClinicalTrials.gov, date of registration is 02/07/2019 and registration numbers is NCT04008745 under the name “Effect of Educational Booklet for Foot-related Exercises for Prevention and Treatment in People with Diabetic Neuropathy (FOCA-II)”. The main trial is currently recruiting, and, for this feasibility study, participants were enrolled from April 23th to September 5th of 2019, totalling 20 weeks of recruitment at the Endocrinology outpatient ambulatory clinic of the Hospital das Clínicas, School of Medicine, University of São Paulo.

The research protocol was published elsewhere^46^. The main trial, if feasible, is designed as a parallel group, *two*-*arm*, superiority *trial with a 1:1 allocation* ratio. All methods were carried out in accordance with relevant guidelines and regulations.

The main trial started and the feasibility study (internal pilot analysis) was carried out with the first 20 enrolled participants from the pre-calculated sample size of the trial. The advantage of this type of design is that it allows us to carry out a prior analysis on the feasibility of recruitment, treatment protocol, adherence, safety, and make amendments (if necessary) without increasing the time required for conducting the complete trial. Thus, the data from participants will be not excluded from final analyses, only if major amendments were necessary to the protocol after the feasibility analysis.

Assessments at baseline (T0) and 8 weeks after the intervention start (T8) were performed at the Physical Therapy department of the School of Medicine, University of São Paulo.

### Participants

A sample size of the first 20 participants of both sexes who were between the ages of 18 and 65 years, had a clinical diagnosis of type 1 or 2 DM has been selected in accordance with published guidance for pilot feasibility studies^47^ who were being treated at the Endocrinology Outpatient Clinic of the Hospital das Clínicas were recruited through telephone contact. Eligibility criteria were DPN score ≥ 2 confirmed by the Decision Support System for Classification of Diabetic Polyneuropathy (http://www.usp.br/labimph/fuzzy)^10^, ability to walk independently for at least 10 m and a maximum of one amputated toe (not being the hallux). Participants were not included if they had an ulcer; a history of surgical procedure to the knee, ankle or hip or indication of surgery or arthroplasty; use of a walking aid such as walker or cane; diagnosis of other neurological disease besides DPN; dementia or inability to give consistent information; were receiving any physiotherapy intervention or using offloading devices during the intervention period; diagnosis of major vascular complications and/or severe retinopathy, as determined from medical files; or had a score of 12-21 (probable depression) on the Hospital Anxiety and Depression Scale. The principal investigator explained to each eligible participant all stages of the study, possible risks, and expected benefits. Upon agreeing to participate, they were asked to sign the Free and Informed Consent Form.

### Randomisation, allocation and blinding

Block randomisation was performed using ClinStat software^47^. The randomisation sequence was organized into blocks with a 1:1 ratio and to prevent blind assessors from predicting allocation, we use more than one block size so that the treatment assignments appear to be more like a simple randomization scheme, and are less likely to be predicted accurately. The block sizes varied randomly between 4 and 8. The randomisation sequence was kept in opaque and sealed envelopes that were numbered sequentially, and the random allocation was performed after acquiring baseline data. An independent researcher not involved with the study assigned the participants to the intervention group (IG; n = 11) or control group (CG; n = 9). Only the physiotherapist responsible for the intervention was aware of group allocation. A physiotherapist and an occupational therapist, both blinded to treatment allocation, were responsible for all clinical and biomechanical outcome assessments. Participants were instructed not to reveal their treatment allocation to the physiotherapists who conducted the assessments. The study statistician was blind to treatment allocation until this preliminary analysis was completed.

### Intervention protocol

CG participants received the usual care, including pharmacological treatment and self-care guidelines of the International Working Group on the Diabetic Foot^14^. All of these usual care orientations were included in a flyer given and explained to the participants of CG and IG by the physiotherapist who conducted the study during the baseline session.

IG participants received the usual care, including pharmacological treatment, plus an 8-week physiotherapeutic home-based foot–ankle exercise programme guided by an educational booklet. This booklet consisted of an informative session and an exercise programme session (see^46^ for more details). The informative session provides information about autonomous footcare, DPN, footwear and the benefits of exercising the foot–ankle. The exercise programme includes six exercises with three difficulty levels. The first exercise session intervention was supervised by the main researcher at the outpatient clinic of the Laboratory of Biomechanics of Human Movement and Posture, School of Medicine, University of São Paulo to instruct the participants on how to perform the exercises using the booklet and to deliver an exercise kit containing materials needed to perform the exercises (cotton balls, a towel, a pencil, mini elastic bands, balloons, light and moderate resistance elastic bands, a massage ball and finger separators). Participants were instructed to perform the exercise programme at home three times a week for 8 consecutive weeks (24 sessions total).

**Figure 4:**
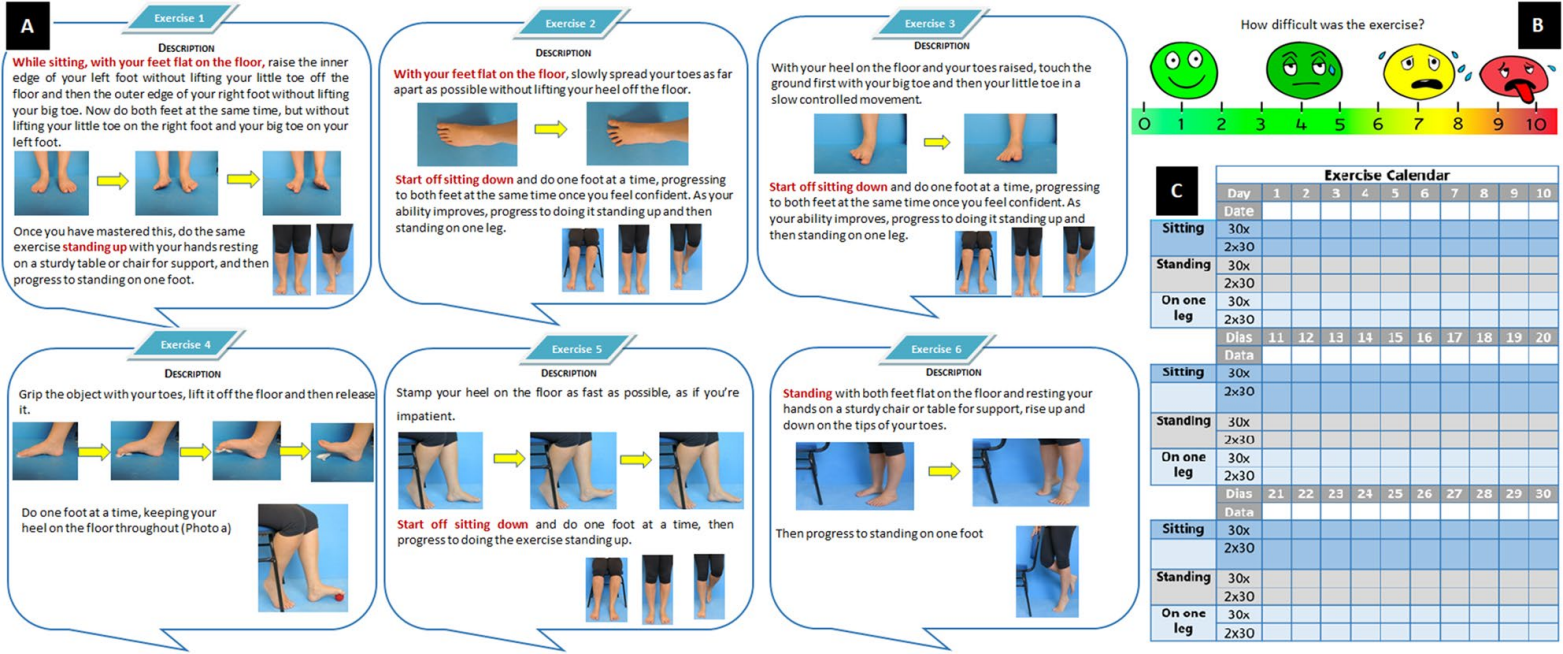
A sample of pages from the booklet. (**A**) exercise programme, (**B**) level of difficulty and (**C**) monthly-calendar. The booklet was created using Adobe Photoshop cs5 Windows (https://www.adobe.com/br/products/photoshop.html) and Microsoft PowerPoint to Microsoft 365 version 2104 and the pictures that make up the figure are from the authors' personal collection.

The physiotherapeutic exercise programme strengthens the intrinsic and extrinsic foot–ankle muscles and increase the ROM of the interphalangeal, metatarsophalangeal and ankle joints. It includes three warm-up exercises, four exercises targeting the intrinsic foot muscles and joints and two exercises targeting the extrinsic foot–ankle muscles and joints (Fig. 4A). Participants could change the difficulty level based on a perceived effort scale (Fig. 4B). The participants were encouraged to record the current difficulty level and number of repetitions of each exercise in the booklet (Fig. 4C). This booklet is an unsupervised exercise programme in which both groups received telephone calls every week from the main researcher to check on their adherence, adverse events, and allowing the participants to take questions about the programme, the disease and the booklet, considering this stage part of the learning process.

### Outcomes

The intervention was considered feasible if participants could be recruited and retained based on the following criteria: (a) participant recruitment rate ≥ to the laboratory availability for assessments (five participants/week); (b) > 75% adherence to the programme^48^; (c) participant dropout ≤ 30%^49^, (d) participant satisfaction with the intervention ≥ 3.75 on a 5-point Likert scale (75% of 5-point scale); and (e) participant safety perception ≥ 3.75 on a 5-point Likert-scale (75% of 5-point scale). Within-group preliminary changes in DPN severity and foot–ankle ROM during gait were assessed for the IG at baseline and T8.

In the full RCT, the primary outcome is the DPN severity given by the fuzzy decision support system. Secondary outcomes are tactile and vibratory sensitivities, foot health and functionality, toe and hallux isometric muscle strength, plantar pressure distribution during gait and foot–ankle kinematics during gait.

The feasibility outcomes were presented for all groups; however, the clinical and biomechanical outcomes for estimating potential changes due to the exercise programme were only reported for IG participants to avoid potential changes in the physiotherapist behavior responsible for providing the interventions, which might affect the reliability of the trial results.

### Feasibility outcomes

#### Recruitment

Recruitment was assessed in terms of contact rate and preliminary screening success as well as recruitment rate. Contact rate is the ratio between the participants contacted at the recruitment period (20 weeks) and the research population universe, expressed in percentage. Preliminary screening success is the ratio between the number of participants who underwent screening and number of the contacted individuals in the recruitment period, expressed as individuals per week. Recruitment rate is the ratio between the eligible participants and the recruitment period.

#### Adherence and dropout

The participants used the monthly-calendar (Fig. 4C) to report the number of sessions they performed and also to describe the exact number of repetitions and sets performed of each exercise (exercise dose). Then, adherence was determined based on data filled in the monthly-calendar regarding number of sessions performed (max 24). The adherence to the home-based programme was the percentage of participants who self-reported completing more than 75% of the 24 sessions (at least 18 sessions) during the 8-week period. The dropout rate was the proportion of participants who completed the therapeutic programme and then dropped out of the study, expressed as a percentage.

#### Participant satisfaction

Participant satisfaction was evaluated at T8 using a seven-item structured questionnaire with a 5-point Likert scale, with higher scores indicating greater participant satisfaction. The questions were: (1) How satisfied were you with the exercise presentation and clarity? (2) How satisfied were you with the opportunity to express your opinion about the booklet? (3) How satisfied were you with the possibility of performing the exercises at flexible and convenient times? (4) How satisfied were you with your feet health after using the booklet? (5) How satisfied were you with the booklet’s ability to encourage the continuation of exercise? (6) What was your general satisfaction with the booklet? (7) How likely are you to recommend the booklet to other individuals with diabetes?

#### Participant safety

In the absence of a systematic instrument to analyse participant safety, safety was assessed at T8 based on the participants’ answer to the question ‘How safe did you feel when performing the exercises at home?’ using a 5-point Likert scale. Furthermore, participants reported any adverse events during weekly calls with the main researcher, as previously recommended^50^. Cramps, moderate to intense pain, fatigue or any other condition that exposed the participant to discomfort were considered.

#### Exercise dose

The exercise dose was measured to assess the feasibility of the protocol in terms of participants’ tolerable exercise volume and intensity. The exercise progression in the booklet is tailored to each participant’s perceived level of effort (Fig. 4B), and the maximum daily volume is six exercises per session with two sets of each exercise and 30 repetitions in each set. The level of difficulty or exercise intensity is based on the posture adopted for the exercises: sitting, standing or standing on one leg. The participants were asked to use the monthly-calendar in the booklet (Fig. 4C) to annotate the exact number of sets and repetitions of each exercise, and the level of difficulty of the exercise (position performed). The exercise volume of each session was the number of exercises performed times the number of repetitions times the number of sets, for a maximum possible total of 360 units [exercise dose = number of exercises (max 6) × number of sets (max 2) × number of repetitions (max 30)]. In each session, general intervention progress was measured as the percentage of participants who performed each posture.

### Clinical and biomechanical outcomes for estimating potential changes

#### DPN severity

DPN severity was defined by the Decision Support System for Classification of DPN^10^ publicly available at http://www.usp.br/labimph/fuzzy. This decision was based on fuzzy logic with the input variables as signs and symptoms extracted from the MNSI, as well as tactile (through the number of non-touch areas using a 10-g monofilament) and vibration sensitivities (measured by vibrating a tuning fork at 128 Hz), characterized as absent, present, or diminished. The fuzzy software gives a score from 0 to 10, with higher scores indicating more severe DPN.

#### DPN symptoms

The Brazilian version of the Michigan Neuropathy Screening Instrument (MNSI)^51^ was used to assess DPN symptoms. Participants answered 15 questions about the sensitivity of their legs and feet. Confirmatory answers for questions 1, 2, 3, 5, 6, 8, 9, 11, 12, 14 and 15 received a score of 1. A negative answer for questions 7 and 13 also scored 1. Question 4 measures circulatory deficit and question 10 measures general asthenia and was excluded from the score. The total score ranged from 0 to 13 (13 representing the worst DPN condition).

#### Foot–ankle ROM during gait

ROM and peak angles from hindfoot relative to tibia (ankle joint), forefoot relative to hindfoot (midfoot motion) and hallux relative to forefoot (first metatarsal joint) in the sagittal plane were assessed by three-dimensional (3D) displacements of passive reflective markers (9.5 mm in diameter) tracked by eight infrared cameras at 100 Hz (VERO, Vicon Motion System Ltd., Oxford Metrics, UK). Forty-two markers were placed on both limbs of the subject (pelvis, thigh, leg, ankle, and foot) according Plug-In Gait and Oxford Foot Model^53^ setup protocols. Participants walked barefoot at a comfortable, self-selected speed on a 10-m walkway. After habituation to the laboratory environment, 10 valid steps on each side were acquired. Automatic digitisation, 3D reconstruction of the markers’ positions and filtering of kinematic data were performed using NEXUS software (version 2.10.3, Vicon Motion System Ltd., Oxford Metrics, UK). Kinematic data were processed using a zero-lag second-order low-pass filter with cut-off frequency of 6 Hz. Kinematics were computed with the open-source Python package pyCGM2 (http://www.pycgm2.github.io) replicating the Vicon Plug-In Gait protocol and Plug-In of Oxford Foot Model.

### Statistical analyses

The present study showed the results for the 20 participants who were recruited, within the predefined recruitment period of 20 weeks. It is recommended that the statistical analysis of any type of pilot or feasibility study should be primarily descriptive^52^ and focus on estimating confidence intervals^53^. There is disagreement regarding whether pilot and feasibility studies should be analysed using hypothesis testing^54^ because it would be inappropriate to assign undue significance, as no formal calculation of power is performed. Because of the small sample, it is likely that there was an imbalance in the pre-randomisation covariates, which would require adjustments to the analysis. In addition, the confidence interval is likely to be inaccurate, even when there are significant differences. The results of any hypothesis test should therefore be treated as preliminary, and analyses within groups should be favoured.

Since this feasibility study consists of a preliminary analysis (internal pilot analysis) of the randomized controlled trial, we chose not to present the CG data at this phase of the trial, in order to avoid possible changes in the behavior of the therapist responsible for providing the interventions, which might affect the trustworthiness of the final results.

Thus, we reported means and standard deviations or medians and interquartile ranges (IQRs) according to data distribution (Shapiro–Wilk test; p > 0.05). In order to not compromise the reliability of the final results of the trial, only the results of the IG are presented and discussed. We estimated the changes in outcomes between baseline and T8 by reporting within-group differences or medians of differences and their 95% confidence intervals.

Due to conceptual problems with using bilateral data from two legs, Menz^55^ recommended against data pooling in foot–ankle research. Because DPN is a symmetrical disease^56^, we chose one side randomly for biomechanical gait analysis (i.e. the left one).

## Data Availability

The datasets used and/or analyzed during the current study are available from the corresponding author on reasonable request.
